# Supplementary material for: Development of CO2-Selective Polyimide-Based Gas Separation Membranes Using Crown Ether and Polydimethylsiloxane
Source: Polymers (Basel). 2021 Jun 10;13(12):1927. doi: 10.3390/polym13121927 (PMC8227709; doi:10.3390/polym13121927)
Supplement: Supplementary file 1 [file polymers-13-01927-s001.zip › polymers-1228636-supplementary.pdf]

<u>Supporting Information for</u>

Development of CO_2 Selective Polyimide-based Gas Separation Membranes Using Crown Ether and Polydimethylsiloxane

Dongyoung Kim,[1,2,⊥] *Iqubal Hossain,*[1,2,⊥]*, Asmaul Husna,*[1,2,⊥] *and Tae-Hyun Kim* [1,2,*]

[1]Organic Material Synthesis Laboratory, Department of Chemistry, Incheon National University, 119 Academy-ro, Yeonsu-gu, Incheon 22012, Korea.

[2]Research Institute of Basic Sciences, Incheon National University, 119 Academy-ro, Yeonsu-gu, Incheon 22012, Korea.

[⊥]These authors contributed equally to this work

*Correspondence: tkim@inu.ac.kr; Tel.: +82-32-8358232

This file includes:

Experimental and measurement

Scheme S1

Figure S1-S7

S1. Experimental and measurement

S1.1. Synthesis of trans-dinitro-DB18C6 (2)

For the synthesis of *trans*-dinitro-DB18C6 (**2**), 5 g of DB18C6 (**1**)(13.87 mmol) was dissolved in 100 mL of HPLC grade chloroform and 75 mL of anhydrous acetic acid. After the DB18C6 was dissolved completely, a mixture of 3.5 mL of HNO_3 with 10 mL of anhydrous acetic acid was added drop wisely over 20 min. The reaction mixture was refluxed at 50 °C for 6 hours, followed by vacuum filtering. The filter cake was washed with chloroform and water to remove unreacted monomer and acetic acid. Then, the white solid was dried under reduced pressure for 24 hours at room temperature. (white solid, 2.73 g, yield 43.4 %); δ_H(400MHz, DMSO-d_6) 7.86(2H, d, *J*=4Hz, *H_a*), 7.69(2H, s, *H_b*),

7.13(2H, d, *J*=4Hz, H_c), 4.19(8H, br, H_d), 3.83(8H, br, H_e); ATR-FTIR (cm^{-1}) 3092(benzene C-H stretching), 2956(C-H stretching), 2886(C-H stretching), 1590(Ph C-C stretching), 1512(N-O asymmetric stretching), 1452(Ph C-C stretching), 1344(N-O symmetric stretching), 1274(Ph-O-C symmetric stretching), 1234(Ph-O-C symmetric stretching), 1136(benzene C-O-C asymmetric stretching)

*S1.2. Synthesis of trans-diamino-DB18C6 (**3**)*

For the synthesis of *trans*-diamino-DB18C6 (**3**), place the 250 mg of Pd/C in the N_2 charged 3-necked flask and pour some ethanol. 2.5 g of *trans*-dinitro-DB18C6 (**2**)(6.22 mmol) was added into the flask with ethanol(100 mL of total ethanol amount), then heated up for 95 °C. After that, 30 mL of hydrazine monohydrate was added drop wisely for 2 hours using a dropping funnel. The mixture solution was stirred overnight at this temperature. The resultant solution was filtered by celite-pad, followed by vacuum evaporating to remove excess hydrazine and ethanol. Ethanol was added to the dried product and wash with a small amount of ethanol several times. White solid was dried under reduced pressure for 24 hours at 80 °C. (white solid, 1.73 g, yield 71.1 %); δ_H(400MHz, DMSO-d_6) 6.60(2H, d, *J*=4Hz, H_a), 6.21(2H, s, H_b), 6.02(2H, d, *J*=4Hz, H_c), 4.61(4H, s, H_f), 3.92(8H, m, H_e), 3.72(8H, m, H_e); ATR-FTIR (cm^{-1}) 3424(N-H stretching), 3456(N-H stretching), 2926(C-H stretching), 2872(C-H stretching), 1592(Ph C-C stretching), 1510(Ph C-C stretching), 1454(Ph C-C stretching), 1280(Ph-O-C symmetric stretching), 1226(Ph-O-C symmetric stretching), 1184(C-N stretching)

S1.3 General analysis technique

All the synthesized monomer structure was confirmed via ^1H NMR spectra obtained on an Agilent 400-MR (400 MHz) instrument using $CDCl_3$ as a reference or an internal deuterium lock. For checking functional group in monomer and membrane, the attenuated total reflection Fourier transform infrared (ATR-FTIR) spectra were obtained using a Perkin-Elmer spectrum two ATR-FTIR spectrometers in the range of 4000-400 cm^{-1}. The surface morphology of the membranes was analyzed by atomic force microscopy (AFM) in tapping mode using a Bruker MultiMode instrument. A silicone cantilever with

an end radius < 10 nm and a force constant of 40 N m^{-1} (NCHR, nanosensors, f=300 kHz) were used to obtain images of samples at ambient temperature. The microstructure of the membranes was also examined by transmission electron microscopy (TEM) on a JEOL JEM-2100 microscope (at an accelerating voltage of 200 kV). The 0.1wt% copolymer solution using chloroform solvent was then subjected to the ultrasonic machine to mix it homogeneously. Then the representative solution was dropped down 1-2 drops on the cupper grid to make a thin homogeneous film, finally, the grid was heated at 75 °C for 12 hours plus 110 °C for 1 hour before taking TEM micrograph. Wide-angle X-ray diffraction (WAXS) patterns of the dry membranes were characterized using a Rigaku HR-XRD Smart Lab diffractometer by employing a scanning rate of 0.2° min^{-1} in a 2θ range from 5° to 30° with a Cu-Kα X-ray (λ = 1.54Å). The dried membranes were placed under vacuum at 60 ºC for 12 h and were equilibrated with 50% RH at least 24 h before the measurement**.**

Gel Permeation Chromatography (GPC) was used to determine the molecular masses. Two PL Gel 30 cm x 5 µm mixed columns at 35 °C were run in $CHCl_3$ and calibrated against Poly(methyl methacrylate) (Mn: 1000 ~ 4•10^6 g mol^{-1}) standards using a refractive index detector.

The thermal stability of the membranes was analyzed by thermogravimetric analysis (TGA) measurements conducted on a SCINCO TGA-1000 instrument in the range of 30-800 °C at a heating rate of 10 °C min^{-1} under a nitrogen flow. The glass transition temperature (T_g) of each crosslinked membrane was measured using a Perkin-Elmer Pyris-1 DSC with a heating rate of 10 °C min^{-1} under a nitrogen flow.

The gas permeation rate is the rate of gas transport across the membrane material and can generally be expressed in terms of the permeance (*Q*) and the permeability (*P*). The permeance is a parameter normalized by pressure and area and is usually employed to characterize asymmetric membranes or thin-film composite membranes. Permeability is the main parameter used to characterize the gas transport rates of dense membranes with a defined thickness, and it is given by the permeance normalized by the thickness (*l*), $P = Q \times l$. More precisely, the gas permeability coefficient (*P)* of a membrane is equal to the pressure and thickness-normalized flux and can be expressed in Eq. (S1).

$$P = \frac{J}{\Delta p} \times l \qquad \text{(S1)}$$

where *J* is the flux of the gas passing through the membrane, *l* is the thickness of the membrane, and

Δp is the permeate pressure rise. The flux of a given gas can be expressed using Fick's first law:

$$J = -D\frac{dC}{dx} \tag{S2}$$

where D is the kinetic parameter, known as the diffusion coefficient, and dC/dx is the concentration gradient of the given gas across the membrane. In the steady state, the flux is a constant, and thus if D is assumed to be constant, Eq. (S2) can be integrated to yield

$$J = -D\left(\frac{C_1-C_0}{dx}\right) = D\left(\frac{C_0-C_1}{l}\right) \tag{S3}$$

where C_0 and C_1 are the gas concentrations in the upstream and downstream regions of the membrane, respectively. At low pressures, the concentration of the gas in the membrane can be expressed with Henry's law:

$$C = S\times p \tag{S4}$$

where S is the thermodynamic factor, known as Henry's solubility constant, and p is the pressure of the gas. By substituting Eq. (S4) into Eq. (S3), we find that

$$J = D\times S\left(\frac{p_0-p_1}{l}\right) = D\times S\left(\frac{\Delta p}{l}\right) \tag{S5}$$

By comparing Eq. (S5) with Eq. (S1), we can see that the gas permeability coefficient (or permeability), P, of the membrane can be defined as the product of the kinetic parameter, that is, the diffusion coefficient (or diffusivity), D, and the thermodynamic parameter, that is, the solubility coefficient (or solubility), S:

$$P = D\times S \tag{S6}$$

In gas separation, the key parameters that characterize membrane separation performance are the permeability coefficient, P, and the selectivity, α. The selectivity is the ratio of the individual gas permeabilities and is also known as the perm-selectivity or ideal gas separation factor. Therefore, for the single gas permeabilities of gas species A and B, the selectivity can be expressed as Eq. (S7).

$$\alpha = \frac{P_A}{P_B} = \frac{D_A}{D_B}\times\frac{S_A}{S_B} \tag{S7}$$

By comparing Eqns (S6) and (S7), we see that the selectivity can also be defined as the product of the diffusivity selectivity and the solubility selectivity. Hence, the gas separation performance of a membrane is determined by the solubility and diffusivity coefficients.

The diffusivity coefficient depends on the mobility of the gas molecules that penetrate through the cavities of the membrane. Several factors influence the gas diffusivity: (i) the size and shape of the gas molecules, (ii) the mobility of the polymer chains, (iii) the cohesive energy density of the polymer, and (iv) the free volume distribution of the polymer. The solubility coefficient refers to the number of molecules dissolved in the polymer matrix. It is affected by: (i) the condensability of the penetrant gases, (ii) the interactions between the penetrant gases and the polymer, and (iii) the chain packing density of the glassy polymers.

Therefore, according to Eq. (S7), the gas separation factor (perm-selectivity, α) of a polymeric membrane can be enhanced by increasing either the solubility selectivity or the diffusivity selectivity through changes to the structure of the polymer. Accordingly, in conventional glassy polymers (high T_g and stiff polymer chains), the diffusivity selectivity dominates over the perm-selectivity, whereas in rubbery polymers (low T_g and flexible polymer chains), the solubility selectivity dominates over the perm-selectivity. However, in contrast to the perm-selectivity, the permeability coefficients (*P*) of glassy and rubbery polymers are governed by the proportion of the diffusivity, and the solubility (Eq. S6) is determined by the physical properties of the membrane matrix, such as the density and the size of its free volume elements.

The gas permeability measurements of the pure gas in the present study were performed using a vacuum-applied time-lag instrument based on a constant-volume/variable-pressure method. All of the experiments were performed at 2 atm of upstream pressure at 30 °C. Before taking these measurements, both upstream and downstream were thoroughly evacuated to below 10^{-5} Torr until the readout showed zero values to ensure the removal of any residual gases. The downstream volume was calibrated using a Kapton membrane and was found to be 57.55 cm^3. The pressures of both sides were measured using a Baratron transducer (MKS; Model No. 626B02TBE) with a full scale of 10,000 and 2 torr, respectively. The permeability coefficient was calculated from the linear slope of the downstream pressure–time plot (*dp/dt*) according to Eq. (S8), which is an elaborated form of Eq. (S1):

$$P = \frac{273}{76} \times \frac{Vl}{ATp_0} \times \frac{dp}{dt} \tag{S8}$$

where *P* is the permeability expressed in Barrer (1 Barrer = 10^{-10} cm^3(STP) $\cdot cm\cdot cm^{-2}\cdot s^{-1}\cdot cmHg^{-1}$), *A* is the effective area of the membrane (cm^2), *V* is the downstream volume (cm^3), *T* is the measurement

temperature (K), l is the membrane thickness (cm), p_o is the pressure of the feed gas in the upstream chamber (torr), and dp/dt is the rate of the pressure change under a steady state. For single gases, the permeability tests were repeated more than three times, and the standard deviation from the mean values of the permeabilities was within ca. ± 3%. The sample-to-sample reproducibility was high and within ± 3%. The effective membrane areas were 15.9 cm^2. The ideal perm-selectivity, $\alpha_{A/B}$, of the polymeric membrane for a pair of gases (A and B) was calculated as the ratio of the individual gas permeability coefficients.

S2. Scheme and Figures

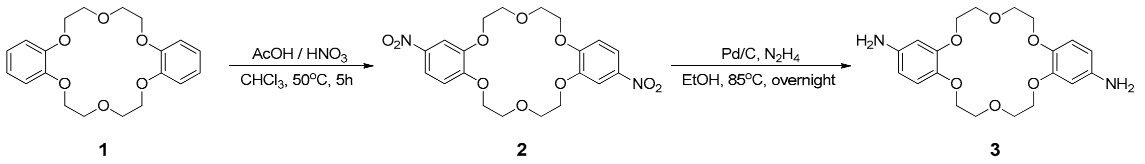

Scheme S1. Synthesis of the diamine-functionalized DB18C6 monomer.

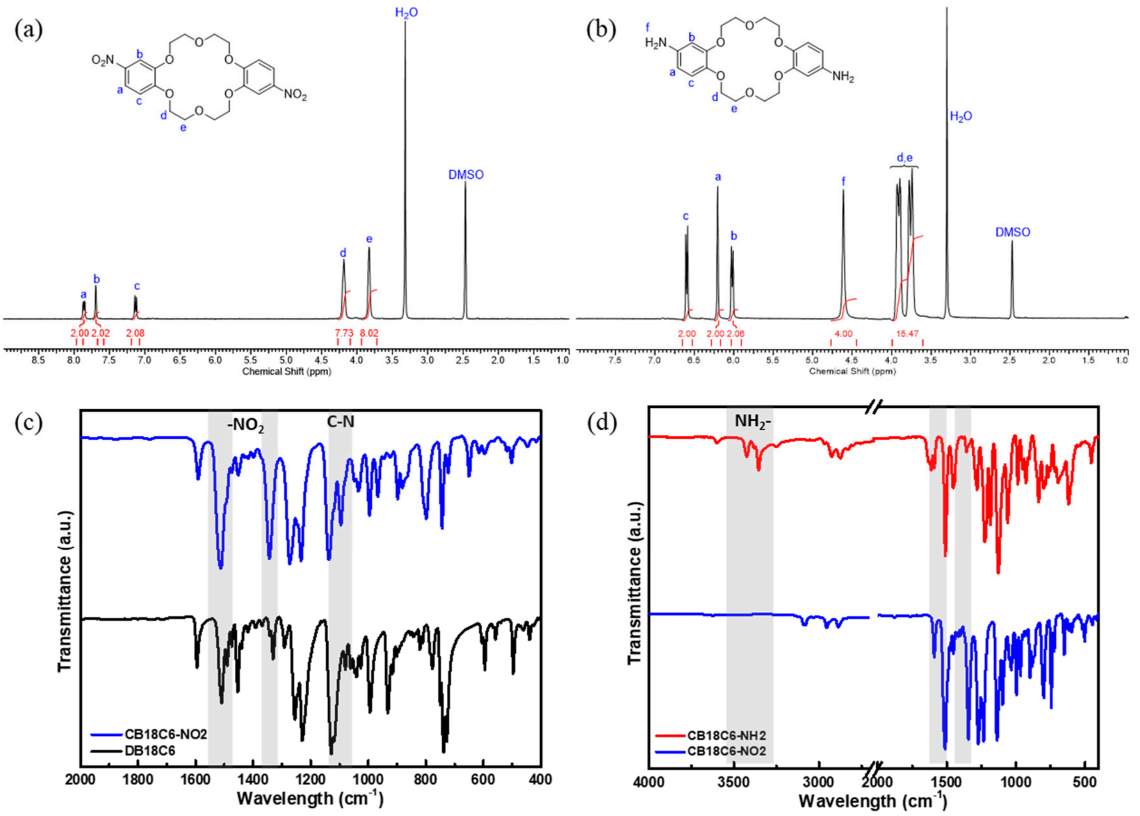

Figure S1. (a), (b) ^{1}H NMR spectra of *trans*-dinitro-DB18C6 **2** and *trans*-diamino-DB18C6 **3** and (c), (d) ATR-FTIR spectra of *trans*-dinitro-DB18C6 **2** and *trans*-diamino-DB18C6 **3**.

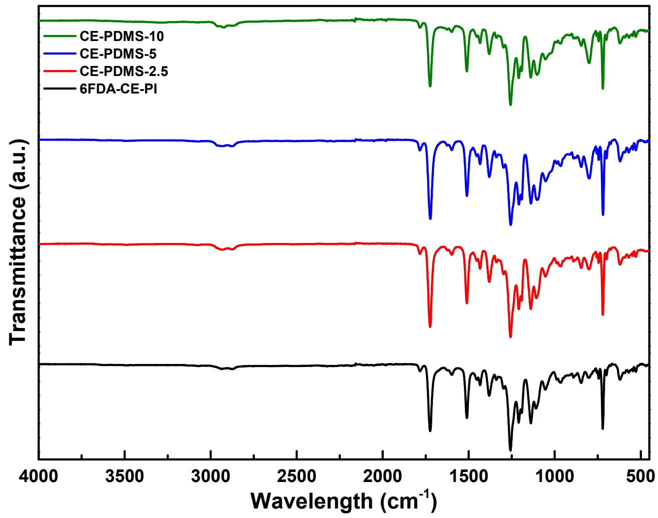

Figure S2. ATR-FTIR spectra of the copolymer membranes with low PDMS content at 4000–500 cm^{-1}

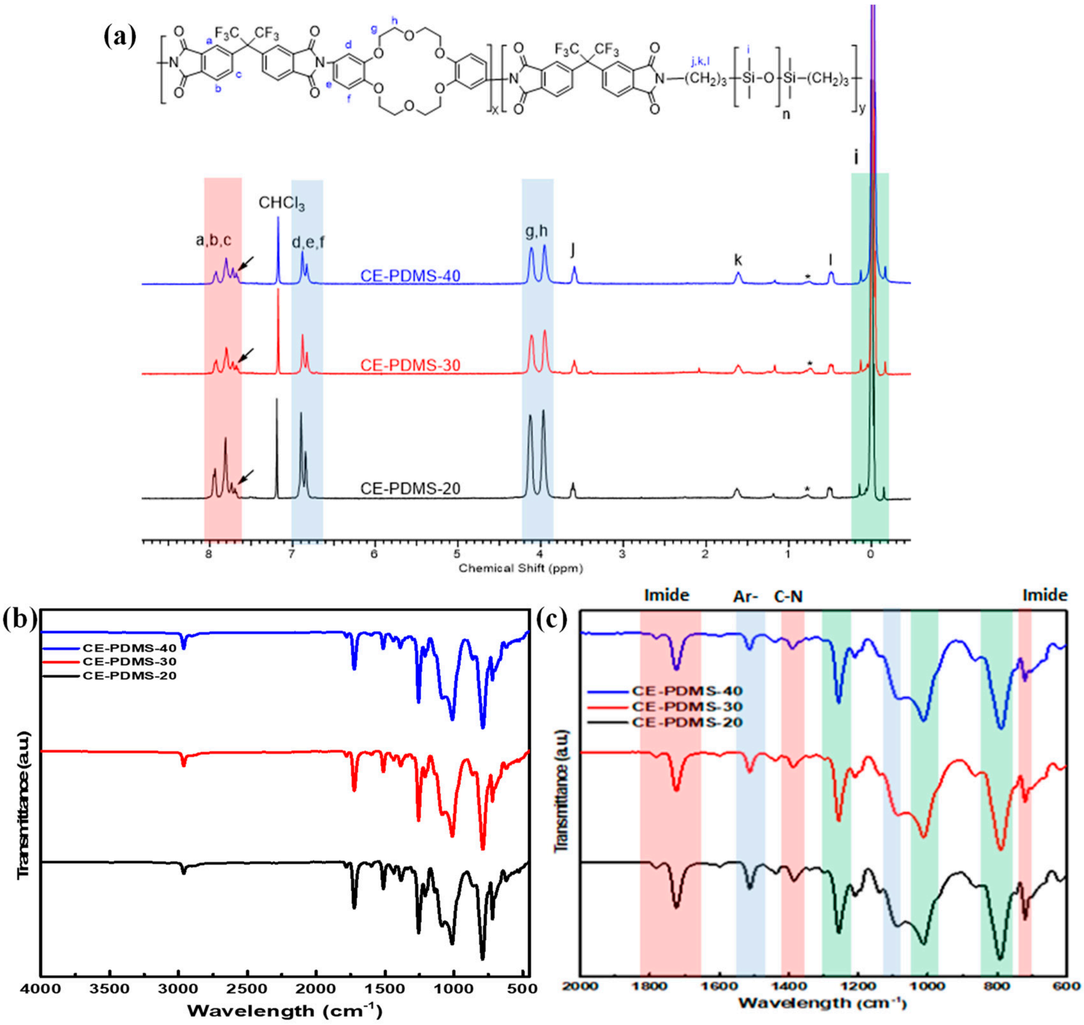

Figure S3. (a) ^{1}H NMR spectra of the high PDMS content membranes and ATR-FTIR spectra of the high PDMS content membranes at (b) 4000 – 450 cm^{-1} and (c) 2000 – 600 cm^{-1}.

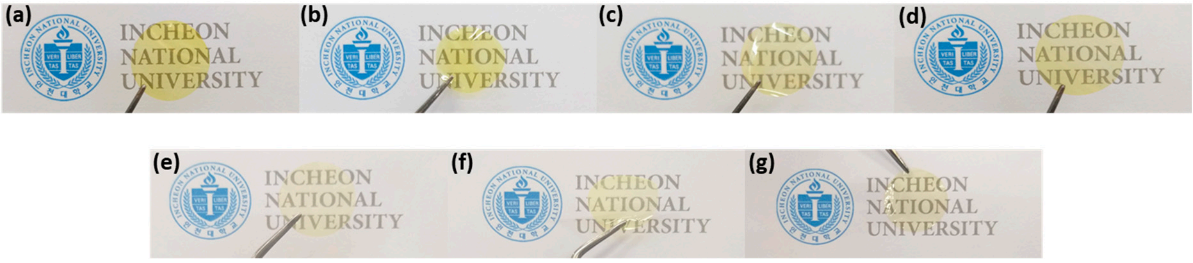

Figure S4. Images of the synthesized polymer membranes (a) 6FDA-CE-PI, (b) CE-PDMS-2.5, (c) CE-PDMS-5, (d) CE-PDMS-10, (e) CE-PDMS-20, (f) CE-PDMS-30, and (g) CE-PDMS-40.

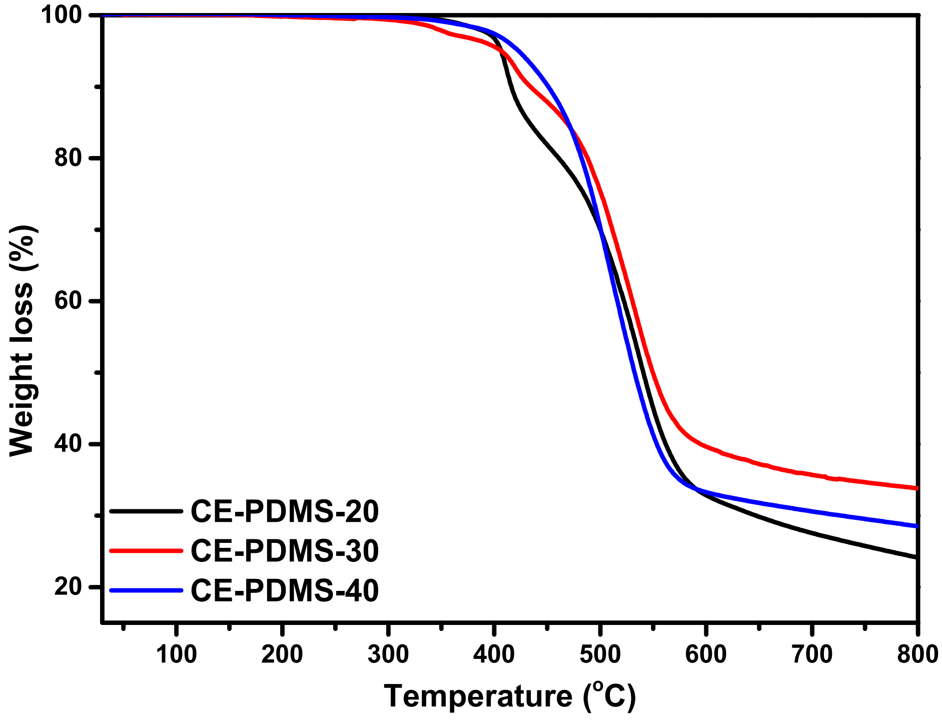

Figure S5. TGA curves of the high PDMS membranes at 30–800 °C

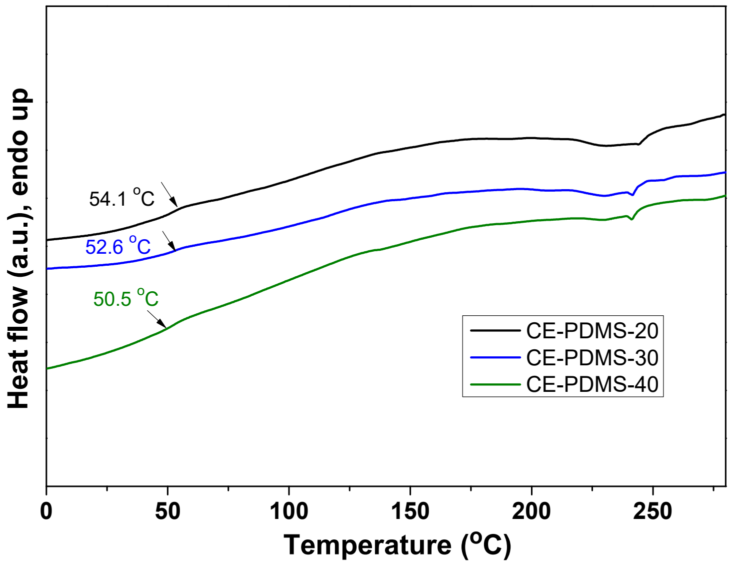

Figure S6. DSC curve and T_g in the CE-PDMS-x membranes loaded with PDMS.

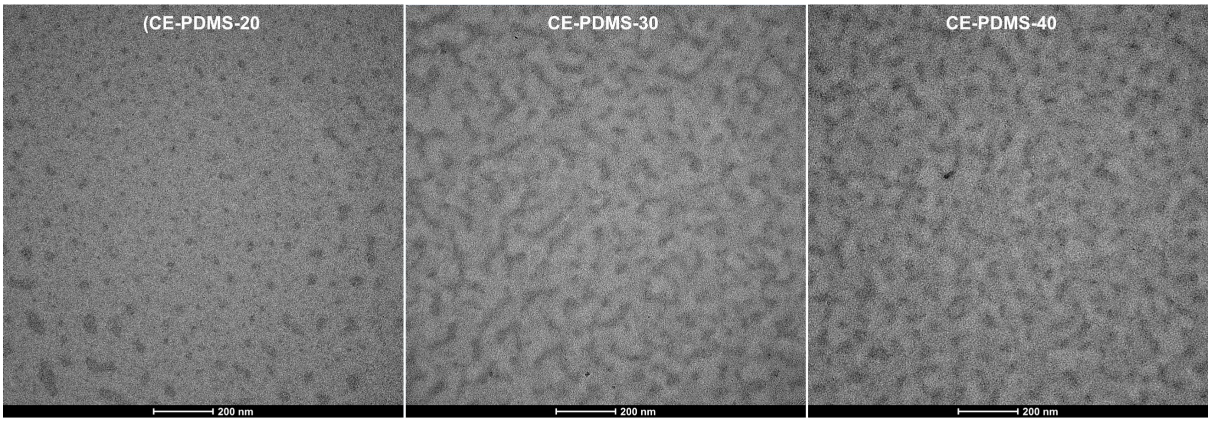

Figure S7. FE-TEM images of the copolymer membranes at high PDMS loadings**.**
